# Maintaining high quality trichiasis surgery before and after trachoma elimination

**Published:** 2023-12-01

**Authors:** Emily Gower, Belay Bayissasse, Amir B Kello, Tim Jesudason

**Affiliations:** 1 Associate Professor, University of North Carolina, Chapel Hill, USA.; 2 Research Project Director, Orbis International Ethiopia, Addis Ababa, Ethiopia.; 3 Medical Officer, Trachoma, World Health Organization Africa Regional Office, Brazzaville, Republic of Congo.; 4 Special Projects and Campaign Partnerships, International Coalition for Trachoma Control, London, United Kingdom.

**Surgical simulation training can help to maintain the quality of trichiasis surgery in a post-elimination setting.**

**Figure:**
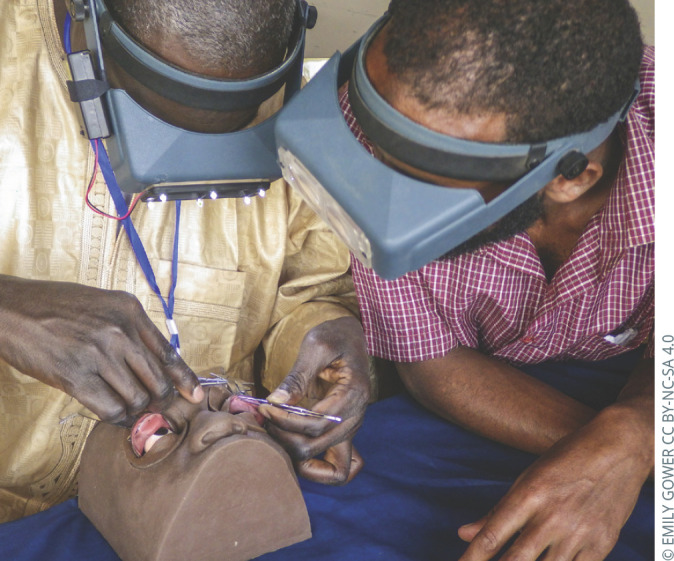
A trainer demonstrates how to perform modified Trabut surgery on HEAD START. **NIGER**

Trachoma, the world's leading infectious cause of blindness, is targeted for global elimination as a public health problem by 2030. The World Health Organization (WHO) criteria for elimination of trachoma as a public health problem are (i) a prevalence of trachomatous trichiasis (TT) unknown to the health system of <0.2% in adults aged ≥15 years and (ii) a prevalence of trachomatous inflammation—follicular (TF) in children aged 1–9 years of <5%, and (iii) evidence that the health system can continue to identify and manage incident cases of TT.^1^

Maintaining the quality of TT surgery – as countries approach and go beyond trachoma elimination – is complicated by the fact that surgeons are conducting fewer operations, which can result in declining surgical skills. Suboptimal surgical quality is not only bad for the patient, but also threatens the achievement of the global elimination of trachoma, as it increases the likelihood that TT will recur, thereby undermining community confidence in trachoma programmes. To maintain the quality of TT surgery, several programmatic activities have been recommended, including certification for surgeons, ongoing supportive supervision, surgeon audits, and refresher training.

*Trichiasis surgery for trachoma*,^2^ the third edition of which will soon be published by WHO, provides a framework for certifying health workers in either bilamellar tarsal rotation or modified Trabut sugery for TT. In order to be certified to carry out TT surgery, health workers must:

1. Complete training in TT surgery in a course of accepted minimum depth and practical content (depending on national policy) and have conducted surgery on at least ten eyelids independently
2. Receive a recommendation for certification from an instructor
3. Successfully perform five sequential operations under observation by the certification examiner, with ‘success’ defined as fewer than 10 unsatisfactory marks on the certification checklist and none in critical areas.

Since the first edition of the WHO manual was published in 1993, it has been used by trainers as a training tool and by surgeons as a reference work, to increase the quality of surgery in settings where trachoma is endemic.

## Surgical simulation

In many trachoma-endemic settings, HEAD START is being used to support trainee surgeons to build their skills and confidence before performing surgery on patients. HEAD START is a surgical simulator on which surgeons can practise surgical skills. The simulator is small and portable, allowing training to take place in remote settings. Previous research has demonstrated that simulation training with HEAD START prior to operating on patients reduces the number of times the trainer needs to intervene when the trainee makes an error and reduces the overall time required for surgery.^3^ HEAD START is also used to provide refresher training and regular professional development for experienced surgeons.

Recent work shows that using HEAD START as part of refresher training for trichiasis surgeons also improves long-term surgical outcomes.^4^ Other work also suggests that the use of HEAD START is readily accepted by surgeons and could be used during periods with low TT surgical activity (such as during the rainy season, when – in many settings – presentations decline).

In many countries, there are not enough trained eye care providers to deliver the surgical services needed to eliminate trachoma as a public health problem, or to ensure that high quality surgical services remain available in a post-elimination setting. HEAD START facilitates the training of general health workers to conduct TT surgery and has the potential to be integrated into tertiary eye health centres. Plans are currently in development to equip secondary and tertiary eye care units in Ethiopia with HEAD START to enable eye care workers to practise their skills. This is essential for the sustainability of programmes and for maximising the impact of limited resources.

The WHO World Report on Vision (bit.ly/world-report-on-vision) emphasises that people who need eye care must be able to receive high-quality interventions. The global trachoma programme provides examples of how continuous professional development systems, including certification and innovative training materials with supportive supervision, can improve the quality of outcomes. However, the trachoma community cannot become complacent. Achieving the elimination of trachoma as a public health problem makes it challenging to maintain quality in post-elimination settings. Doing so requires forward thinking to ensure that trachoma interventions, including training, supervision, and certification, are integrated into routine health systems.
